# Bevacizumab to Treat Cholangiopathy in Hereditary Hemorrhagic Telangiectasia: Be Cautious: A Case Report

**DOI:** 10.1097/MD.0000000000001966

**Published:** 2015-11-20

**Authors:** Quentin Maestraggi, Mohamed Bouattour, Ségolène Toquet, Roland Jaussaud, Reza Kianmanesh, François Durand, Amélie Servettaz

**Affiliations:** From the Department of Internal Medicine, Infectious diseases and Clinical Immunology, Robert-Debré Hospital, Université de Reims Champagne-Ardenne, Reims, France (QM, ST, RJ, AS); Department of Hepatology and Liver Intensive Care, Assistance Publique-Hôpitaux de Paris, Clichy, France (MB, FD); and Department of General, Digestive and Endocrine Surgery, Robert-Debré Hospital, Université de Reims Champagne-Ardenne, Reims, France (RK).

## Abstract

Hereditary hemorrhagic telangiectasia (HHT) is an inherited vascular dysplasia characterized by mucocutaneous telangiectasia and visceral arteriovenous malformations. Hepatic involvement with vascular malformations may lead to portal hypertension, biliary ischemia, and high-output cardiac failure. There is no curative treatment for the disease. Liver transplantation is indicated for life-threatening complications, but it carries significant risk due to surgery and immunosuppressive treatment. Some case reports or small open studies suggest that bevacizumab, a recombinant humanized anti-VEGF monoclonal antibody, should be efficient in limiting bleeding and in reducing liver disease in HHT.

We report a case of a 63-year-old woman with HHT presenting with ischemic cholangiopathy. Liver transplant was indicated, but given a previous encouraging report showing a regression of biliary disease with bevacizumab in 3 patients with HHT this drug was proposed. No significant efficacy but a severe adverse effect was observed after 3 months: bilateral pulmonary embolisms, thrombosis in the right atrial cavity, and thrombosis of the right hepatic vein were evidenced. Bevacizumab was stopped; anticoagulant started. Four months later, the patient received a transplanted liver. She feels well 1 year later.

This case report intends to provide the information for clinicians to consider the use of bevacizumab in HHT. Whereas several uncontrolled series and case reports have suggested the efficacy of this drug in reducing bleeding and liver disease, no severe side effects were mentioned to date. For the first time in HHT we report a life-threatening side effect of this drug and no efficacy. Moreover, systemic thrombosis, the observed complication, may preclude transplantation. To date, caution seems still indispensable when considering the use of bevacizumab in HHT.

## INTRODUCTION

Hereditary hemorrhagic telangiectasia (HHT) (also known as the Osler–Weber–Rendu syndrome) is an autosomal dominant vascular dysplasia characterized by mucocutaneous telangiectasia and visceral arteriovenous malformations in virtually any organ. Hepatic vascular malformations are common, though rather asymptomatic. Symptomatic liver diseases may present high-output cardiac failure, portal hypertension, or ischemic biliary disease.^1,2,3^ Such complications are potentially fatal without liver transplantation.

Few medical therapies are efficient in HHT. As vascular endothelial growth factor (VEGF) levels have been shown to be ∼15-fold elevated in patients with HHT bevacizumab, a recombinant humanized anti-VEGF monoclonal antibody, has been used in some patients.^4^ Several uncontrolled series have reported the efficacy of this drug in reducing epistaxis and iron deficiency related to HHT.^5,6,7^ Some case reports and only 1 single-center phase 2 trial have suggested that intravenous bevacizumab should also be a promising medical option in HHT symptomatic liver disease.^8,9,10,11^ These few reports on the use of intravenous bevacizumab to treat complicated liver vascular malformations have never mentioned severe side effects. Nevertheless, bevacizumab exposes to the risk of important systemic side effects, and its efficacy both on bleeding and on liver disease has not yet been demonstrated.

We report the case of 1 patient with ischemic cholangiopathy due to liver vascular malformations. Given the results reported in 3 HHT patients with similar biliary disease intravenous bevacizumab was proposed. The patient developed severe thromboembolic complications and no improvement was observed in the liver disease.

## CASE REPORT

A 63-year-old woman was admitted on April 2013 with a 15-day history of right upper quadrant pain and fever for a week. She had a known definite clinical diagnosis of HHT based on the presence of all 4 Curaçao criteria (diagnosis of HHT in a first degree relative, recurrent spontaneous epistaxis, multiple typical mucocutaneous telangiectasia, and hepatic vascular malformation), and she had been confirmed to harbor the known familial mutation within the intron 5 of the ALK1 gene.

**FIGURE 1:**
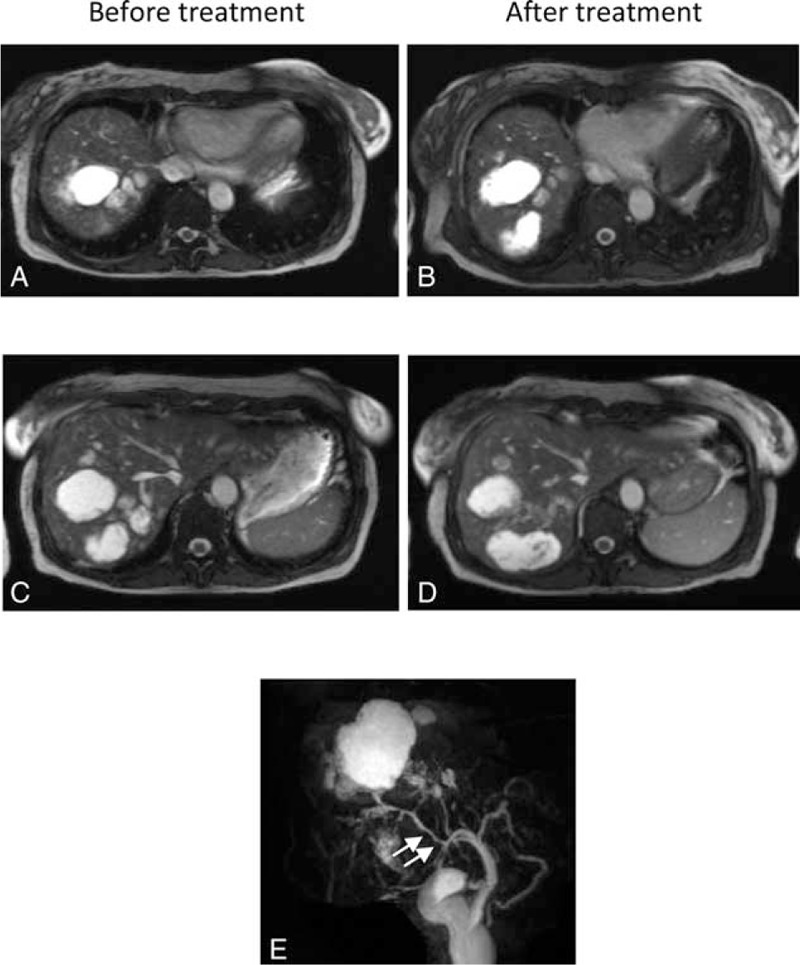
Liver MRI images before and after bevacizumab treatment. (A, B) axial balanced fast-field echo-weighted MRI images obtained before bevacizumab treatment demonstrate multiple bilomas. (C) MR cholangiography images show multiple areas with irregularities of the bile ducts arrows. (D, E) after 6 courses of bevacizumab, liver MRI images revealed an increase in the size and number of bilomas and persistence of the biliary irregularities. MRI = magnetic resonance imaging.

Nine years before she had presented *Staphylococcus aureus* bacteremia with hip osteoarthritis. The front door of *Staphylococcus* had not been clearly identified, but the nose was suspected because of her recurrent epistaxis due to HHT. Hepatic vascular malformations had been discovered by echography with Doppler performed as recommended for the follow-up of HHT in 2006. They had remained stable on CT, asymptomatic, and no abnormality in the cardiac output had been detected until then. She had never presented any other clinical complication of HHT but had chronic sicca syndrome related to the Sjögren syndrome. She had since been managed with iron and artificial tears. Physical examination revealed hepatomegaly without abnormal cardiac murmur. Laboratory analyses demonstrated moderate anemia (hemoglobin 105 g/L), multiple positive blood cultures for *Staphylococcus aureus* and cholestasis (Table 1). Transthoracic and transesophageal cardiac echography showed normal biventricular size, valves integrity, and normal cardiac index. Thoracic and cerebral computed tomography (CT) was normal without abscess or vascular malformation. Liver MRI revealed arterio venous shunting and intrahepatic bilomas. The patient was treated intravenously with oxacillin and gentamicin for 2 weeks, then with oral amoxicillin, clavulanic acid, and ciprofloxacin for 6 weeks. The front door of *Staphylococcus* was not clearly identified, but the nose was suspected because of recurrent epistaxis. Fever and bacteremia resolved but the patient continued to suffer from paroxysms of pain after eating, despite analgesia, and from a progressive weight loss. No sign of hip osteoarthritis was evidenced. Cholestasis increased (Table 1) and liver MRI demonstrated diffuse bile duct necrosis and intrahepatic bilomas (Figure 1A–C).

**TABLE 1:**
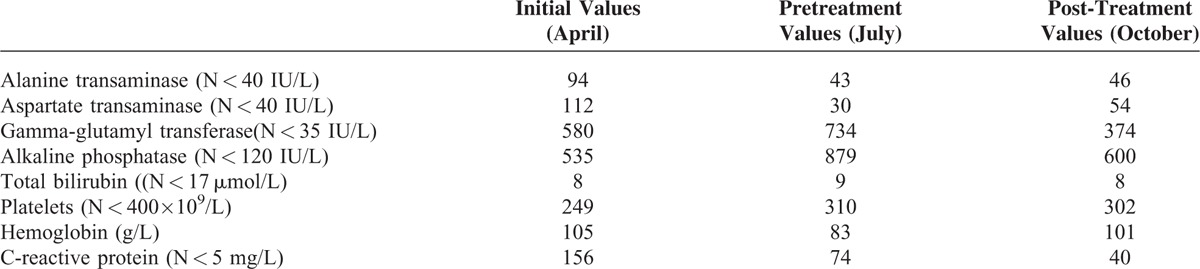
Laboratory Findings Before and After Therapy

Considering the severity of the presentation and the recently reported cases of the efficacy of bevacizumab in HHT cholangiopathy, the patient was started on bevacizumab with the published protocol (5 mg/kg intravenously at 2-week intervals for a total of 6 doses).^8^ During these 3 months abdominal pain decreased but did not totally disappear. Her ECOG performance status was grade 1. She was discharged and busied herself with bed and breakfasts. No hypertension, proteinuria, or renal insufficiency was observed; laboratory evaluation showed a mild decrease of cholestasis liver enzymes (Table 1).

**FIGURE 2:**
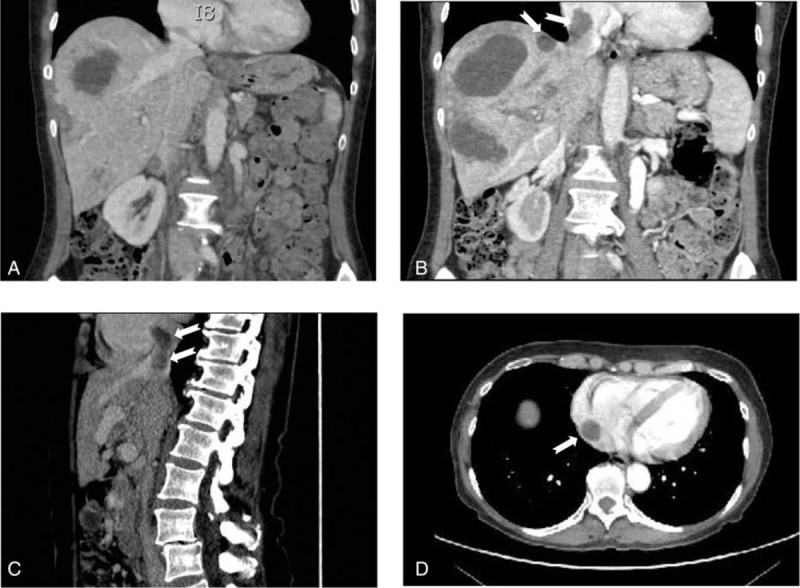
A thoracic and abdominal CT was performed before (A) and after 6 courses of bevacizumab (B, C, D). A (coronal reformatted CT): pretreatment imaging demonstrates multiple hypodense lesions in keeping with bilomas and permeable hepatic veins and inferior vena cava. B, C, D: following treatment, bilomas remain unchanged and thrombosis in the right hepatic vein (B, C), in the inferior vena cava (C) and in the right atrial cavity (D) were evidenced (arrows). CT = computed tomography.

After 6 courses of bevacizumab a programmed thoracic and abdominal CT showed bilateral pulmonary embolisms, thrombosis in the right atrial cavity and thrombosis of the right hepatic vein (Figure 2). The patient had neither dyspnea nor sign of right heart failure. Her ECOG performance status was grade 1. Liver CT and MRI revealed an increase of the biloma size (Figure 1D and E). Liver MRI also demonstrated irregular intrahepatic bile duct necrosis with biloma. Bevacizumab was stopped, anticoagulant was started, and the patient was listed for liver transplantation. During this period the patient continued to suffer from pain paroxysms after eating, but her weight remained stable. The patient was transplanted 4 months later. Liver CT performed before transplantation still demonstrated irregular intrahepatic bile duct necrosis with biloma. The biloma sizes were similar to those measured 4 months earlier. The pathological evaluation of hepatic explant showed several thromboses of hepatic and portal veins and cystic dilatations secondary to ischemic cholangiopathy. No evidence of fatty liver disease was observed. She remains well 1-year following transplantation.

## DISCUSSION

VEGF is a key regulator of angiogenesis and serum; tissue expression of VEGF is increased in HHT patients. By inhibiting the VEGF pathway bevacizumab has raised hope in different complications of HHT for the past few years.^4^

Several uncontrolled series have suggested the efficacy of this drug in reducing epistaxis and iron deficiency related to hemorrhagic telangiectasia.^5,6,7^ Most of the published articles consist in case reports and have reported a reduction of epistaxis or gastrointestinal bleeding by using 5 mg/kg intravenous bevacizumab every other week for 1 to 3 months. In all reported cases, in which a follow-up was mentioned, bevacizumab effects were always transient, and retreatment was proposed after a few months. No severe side effect was mentioned in these few cases.

Four case reports and only 1 single-center phase 2 trial have suggested that intravenous bevacizumab may also be a promising medical option in HHT symptomatic liver disease.^8,9,10,11^ In 2008, Mitchell et al reported the case of a 47-year-old woman with liver arteriovenous malformations complicated by high-output cardiac failure and portal hypertension.^11^ Six courses of intravenous bevacizumab (5 mg/kg) induced a rapid clinical improvement, a decrease of hepatic vascularity and normalization of cardiac output. This status was stable at the 6-month follow-up visit when the case was published. In 2012, Dupuis-Girod et al designed a noncomparative single-center phase 2 trial to assess the efficacy of bevacizumab (5 mg/kg every 14 days for a total of 6 intravenous injections) in reducing cardiac output in HHT hepatic forms.^9^ Among 23 patients with data at 6 months, cardiac output was normalized in 5 and decreased in 15. One patient developed a severe side effect with systemic hypertension, and 20 patients had at least 1 adverse effect possibly related to bevacizumab. No patient experienced thromboembolic event. Chavan et al reported the efficacy of the same protocol of bevacizumab in 3 patients with symptomatic liver complications.^10^ Two of them had initially a high cardiac output that was decreased by the treatment. A third patient with a normal cardiac output was improved in terms of fatigability. No side effect was mentioned. Vlachou et al reported in 2013 3 patients with ischemic cholangiopathy, whose clinical and radiological responses were obtained by the previously given protocol of bevacizumab: 5 mg/kg intravenously at 2-week intervals for a total of 6 doses followed by a maintenance dose, without adverse effect during a 1-year follow-up.^8^

Our patient presented with ischemic cholangiopathy with superimposed infection, as this was the case in 2 patients treated by Vlachou and colleagues.^8^ We applied the same treatment protocol, but no significant improvement of the biliary disease was observed. Moreover, our patient developed extended thrombosis of the hepatic vein and pulmonary embolisms. Indeed, arterial and venous thromboembolisms are a known possible adverse effect of anti-VEGF molecules. In a meta-analysis including 15 randomized controlled trials and 7956 patients with cancer the patients treated with bevacizumab had a significantly increased risk of venous thromboembolisms.^12^ Nevertheless, this data is drawn from studies in patients with malignancies; the risk may be different in other diseases, such as HHT.

Our patient had no history of malignant lesion, and clinical and morphological examination did not reveal any at the time of the event and after >1-year follow-up. She had no history of personal or familial thrombosis. Nonalcoholic fatty liver disease (NAFLD) has been reported in patients with HHT, and this condition has been shown to be independently associated with idiopathic venous thromboembolism in the general population.^13,14^ Regarding our patient, careful examination by pathologists of the hepatic explant did not show any fatty liver disease. Therefore, NAFLD cannot be considered as a cause of venous thromboembolism for this case. An increased risk of developing deep vein thrombosis has been evidenced in patients with Sjögren syndrome by Chung and colleagues in the national cohort in Taiwan.^15^ Our patient had been diagnosed with Sjögren syndrome many years earlier. She had never experimented deep venous thrombosis, even during her prolonged immobilization for hip arthritis. So, Sjögren syndrome is not the main explanatory factor of thrombosis in her case. Nevertheless, this condition cannot be excluded, for it may have favored the development of thrombosis. Our patient was hospitalized only a few hours for her intravenous injections, and her ECOG performance status was grade 1; so we believe that her thromboembolism event was mainly due to bevacizumab injections. We also hypothesize that the local hepatic inflammation and the likely infection of biloma have been major determinants for the localization of the hepatic veins thrombosis.

In addition to thrombosis intravenous bevacizumab exposes to the risk of other severe systemic side effects: hypertension, increased bleeding risk, mucosal perforation, proteinuria, and thrombotic microangiopathy. Severe hypertension was reported in a patient with HHT treated with bevacizumab for high cardiac output, and Dupuis-Girod et al reported frequent moderate side effects in 20 among their 25 treated patients (headache, nausea and vomiting, asthenia, diarrhea, and pain).^9^ All patients received the same dose (5 mg/kg) and the factors involved in the outcome of one or more side effect(s) were difficult to be unraveled to date. Regarding our patient we strictly applied the same protocol as described by Vlachou et al for patients with HHT and ischemic cholangiopathy.^8^ Our patient's clinical examination was performed every 2 weeks before perfusion with a control of blood pressure, blood, and urinary analyses. During these 3 months abdominal pain decreased but did not totally disappear; no hypertension, proteinuria, or renal insufficiency was observed; laboratory evaluation showed a mild decrease of cholestasis liver enzymes. Our patient had neither dyspnea nor sign of right heart failure; otherwise, thoracic CT and echocardiography would have been performed previously. Bilateral pulmonary embolisms, thrombosis in the right atrial cavity, and thrombosis of the right hepatic vein were evidenced while she was asymptomatic. Given this experience we propose to use cautiously bevacizumab in HHT. Further studies concerning the use of bevacizumab in ischemic cholangiopathy should be performed in HHT patients, including patients that do not need liver transplantation. Furthermore, if this drug is finally proposed thoracic and abdominal CT should be performed earlier, after 4 doses. Besides, Doppler of hepatic and portal veins could be suggested before treatment and immediately in cases of refractory abdominal pain, febrile episodes or worsening of liver biochemistry during the course of the treatment.

A few cases have recently reported the efficacy of low (2–3 mg/kg) or very low dose (0.125 mg/kg) of intravenous bevacizumab to limit bleeding with no or moderate side effect.^16,17^ A nasal spray containing this antiangiogenic molecule is also currently on trial to reduce epistaxis in HHT.^18^ The effect of this nasal spray on systemic complications has not been investigated yet. However, the systemic diffusion of the molecule seems very limited after intranasal delivery; so the spray should mainly be efficient on epistaxis.

## CONCLUSIONS

Currently, given the small number of reported patients with HHT treated by bevacizumab, the large spectrum of the visceral involvement observed in HHT, and given the possible severe adverse effect of the drug it seems important to use cautiously bevacizumab in HHT. Its efficacy in each type of complication of HHT, its dosage and its safety should be determined in a large multicenter randomized study.

This observation shows that patients with HHT receiving bevacizumab may develop systemic thrombosis, a complication that may preclude transplantation.
